# Exploring the dynamics of stroke survivors’ care dependency, fatigue, and coping strategy in their caregivers: a structural equation modeling analysis

**DOI:** 10.3389/fpubh.2025.1528109

**Published:** 2025-05-21

**Authors:** Jinyao Wang, Shuangyan Tu, Jun Cui, Rong Yang, Yan Jiang, Lihong Zhao

**Affiliations:** ^1^West China School of Nursing, Sichuan University/Department of Cardiology, West China Hospital, Sichuan University, Chengdu, China; ^2^Department of Neurology, West China Hospital, Sichuan University/West China School of Nursing, Sichuan University, Chengdu, China; ^3^Department of Infrastructure, West China Hospital, Sichuan University, Chengdu, China; ^4^Department of Nursing, West China Hospital, Sichuan University/West China School of Nursing, Sichuan University, Chengdu, China; ^5^Department of Radiology, West China Hospital, Sichuan University/West China School of Nursing, Sichuan University, Chengdu, China

**Keywords:** stroke, care dependency, caregiver fatigue, coping strategy, SEM

## Abstract

**Aims:**

To evaluate the interactions among stroke survivors’ care dependency, caregivers’ negative coping strategy, and fatigue.

**Design:**

Descriptive quantitative research design.

**Setting:**

Patients with acute stroke and their caregivers were enrolled in a General Hospital in West China from July 2022 to April 2023.

**Primary and secondary outcome measures:**

Data collection adopted the Care Dependency Scale (CDS), the Simple Coping Style Questionnaire (SCSQ), and the Fatigue Scale-14 (FS-14). Structural equation modeling was applied to test the hypothetical model. This study adhered to STROBE reporting guidelines.

**Results:**

A total of 380 dyads with the mean age of stroke caregivers was 63.41 ± 12.87 years. The mean score for care dependency, fatigue, positive coping, and negative coping were 53.91 ± 15.95, 8.34 ± 3.58, 19.39 ± 7.05, and 8.06 ± 4.23, respectively. The structural equation model had a good fit index (χ^2^ = 12.595 (*df* 5), IFI = 0.993, GFI = 0.990, AGFI = 0.956, NFI = 0.988, TLI = 0.979, CFI = 0.993, RMSEA = 0.063, SRMR = 0.0213). It showed that the direct effect path coefficients of patients’ care dependency on caregivers’ fatigue (β = −0.22, 95% confidence interval (CI): −0.367, −0.097, *p* = 0.002) and negative coping (β = −0.31, 95% CI: −0.438, −0.155, *p* = 0.003) were statistically significant. The NIHSS score indirectly influenced caregivers’ fatigue (β = 0.023, 95% CI: 0.006, 0.051, *p* = 0.002) and negative coping (β = 0.204, 95% CI: 0.103, 0.303, *p* = 0.003) through the mediating effect of care dependency.

**Conclusion:**

This study indicated that stroke patients’ level of care dependency directly impacts their caregivers’ negative coping strategy and level of fatigue. The stroke’s severity might induce caregivers to engage in negative coping strategies and endure more fatigue when the patients’ care dependency played an indirect role. Researchers need to develop targeted interventions involving training caregivers to employ active coping strategies to confront the care burden.

**Implications for the profession and/or patient care:**

Based on the treatment of stroke onset, encouraging and training caregivers to adopt active coping strategies has the potential to pave the way to relieve care burden by reducing caring fatigue and improving the care quality.

**Impact:**

Addressing stroke caregivers’ common fatigue highlights the importance of optimizing the recovery process to alleviate patients’ care dependency through better treatment and rehabilitation.

## 1 Introduction

Stroke is a leading cause of death and a significant cause of adult disability worldwide. About 15 million people across the world suffer strokes each year (1–3). As reported, China experiences 3.3 million new stroke cases every year, and it is estimated that the number of stroke patients will be 31 million by 2030. In China, 1.54 million people die from stroke annually, while approximately 80% of stroke survivors have disabilities of varying degrees (4, 5). Stroke has emerged as the third highest cause of death and the leading cause of disability-adjusted life-year (DALY) in China in 2019 (6). For acute ischaemic stroke, the reported case fatality increases from less than 5% at 1 month to 10% at 3 months and 15% at 1 year (7).

The majority of stroke survivors required assistance to perform daily living activities and experienced dependency on nursing care in the long term (8, 9). Care dependency in stroke survivors is associated with the fulfillment of basic multidimensional needs. It is subject to change over time, which places caregivers who provide direct care at risk of neglecting their own health needs and becoming hidden patients (10). The continuous caregiving burden accumulated from this dependency can result in negative emotional, social, environmental, and health-related difficulties for both stroke survivors and their caregivers (11). Notably, this burden is increasingly recognized as overwhelming for caregivers (12–15). Therefore, the caregivers of disabled stroke survivors warrant urgent attention, as supporting their critical role is essential to improving outcomes for all involved.

## 2 Background

Generally, clinical fatigue can be classified as either objective or subjective; objective fatigue is defined as the observable and measurable decrement in performance occurring with the repetition of a physical or mental task, while subjective fatigue is a feeling of early exhaustion, weariness, and aversion to effort. Post-stroke fatigue is generally thought to be a primary fatigue (16). It is regarded as overwhelming exhaustion or tiredness, differing from sadness or weakness and typically not improved by rest (17, 18). The worldwide prevalence of post-stroke fatigue varies from 23 to 77%, depending on different definitions and measurements (19). From both stroke survivors and their caregivers’ perspectives, this fatigue is highly linked with poor activities of daily living, manifesting as limitations in physical activities or restricted social participation, leading to lower health-related quality of life. Notably, women in caregiving roles are more likely to experience stress and other adverse health impacts like fatigue compared to men (20). In Chinese societies, caregivers’ fatigue stems not only from the heavy burden and distress of caregiving activities but also their perception of marginalization by primary healthcare services due to the limited availability of information on stroke recovery and delayed or inadequate healthcare services (21). Prior research suggests that a more profound understanding of post-stroke fatigue might enable healthcare professionals to identify caregivers experiencing early objective fatigue and who could benefit from further support easily (17, 22). However, little is known about factors improving caregiver post-stroke fatigue or the personal coping strategies used by caregivers with lived experience of post-stroke fatigue (23).

Coping is a process that addresses how people respond and act when experiencing stress and when the level of exposure to stress rises. The ways of coping are mainly identified as problem-focused coping (including confronting coping and planful problem-solving) and emotion-focused coping (including distancing, self-controlling, accepting responsibility, positive reappraisals, and escape-avoidance) (24). Problem-focused and adaptive coping strategies are likely to play a protective role in relieving caregivers’ burden (25). For example, a strength-oriented psychoeducational program that aims to enhance caregivers’ problem-solving and coping abilities showed a significantly lower level of caregivers’ burden 3 months after the intervention (26). On the contrary, stroke caregivers who do not feel they have the coping ability to face a particular stressful situation probably adopt ineffective coping strategies. They may adopt escape-avoidance thoughts or behaviors to escape stressful situations or problems, which have negative implications for stroke survivors being cared for (27). The caregivers’ escape-avoidance coping also leads to increasing caregiving burden and post-stroke caregiving fatigue as a prolonged issue to fight (28).

Lazarus and Folkman’s Transactional Model of Stress and Coping theory, the most well-established theory for coping with stressful situations, serves as our framework basis. Their coping concept is process-oriented rather than trait-oriented, it discusses the relation between coping strategy and individuals’ health outcomes with time. Throughout this entire process, the coping strategies, which are manifested in how to utilize and allocate individuals’ limited social, physical, and psychological resources to deal with stressful situations, play an essential role in modulating the physical and psychological burden (24, 29). Positive coping (e.g., problem-focused coping) tended to be associated with better physical and psychological functioning, whereas the use of negative coping (e.g., emotion-focused coping) had adverse effects on physical and psychological functioning. Evidence indicates that applying flexible and positive coping strategies including taking measures to alleviate patients’ care dependence, which is significantly correlated with stroke patients’ severity, has a positive impact on the adverse outcomes of both patients and their caregivers (30).

In this regard, a structural model was developed to elucidate and predict caregiver fatigue in stroke survivors by verifying the fit of the hypothesized model. Our study set out to estimate the effects of clinical variables (stroke patients’ severity, patients’ care dependency, and caregiver characteristics) and personal response (caregivers’ coping strategy) on caregivers’ outcomes (post-stroke fatigue). We hypothesized that the care dependency of stroke survivors affects caregivers’ fatigue directly and indirectly through the intervening effect of their negative coping strategy. The secondary hypothesis is that the stroke patients’ severity directly affects caregivers’ fatigue, and their relationship also could be indirectly regulated by the effects of negative coping strategy and care dependency of stroke survivors.

## 3 Methods

### 3.1 Aims and hypothetical model

**Figure 1:**
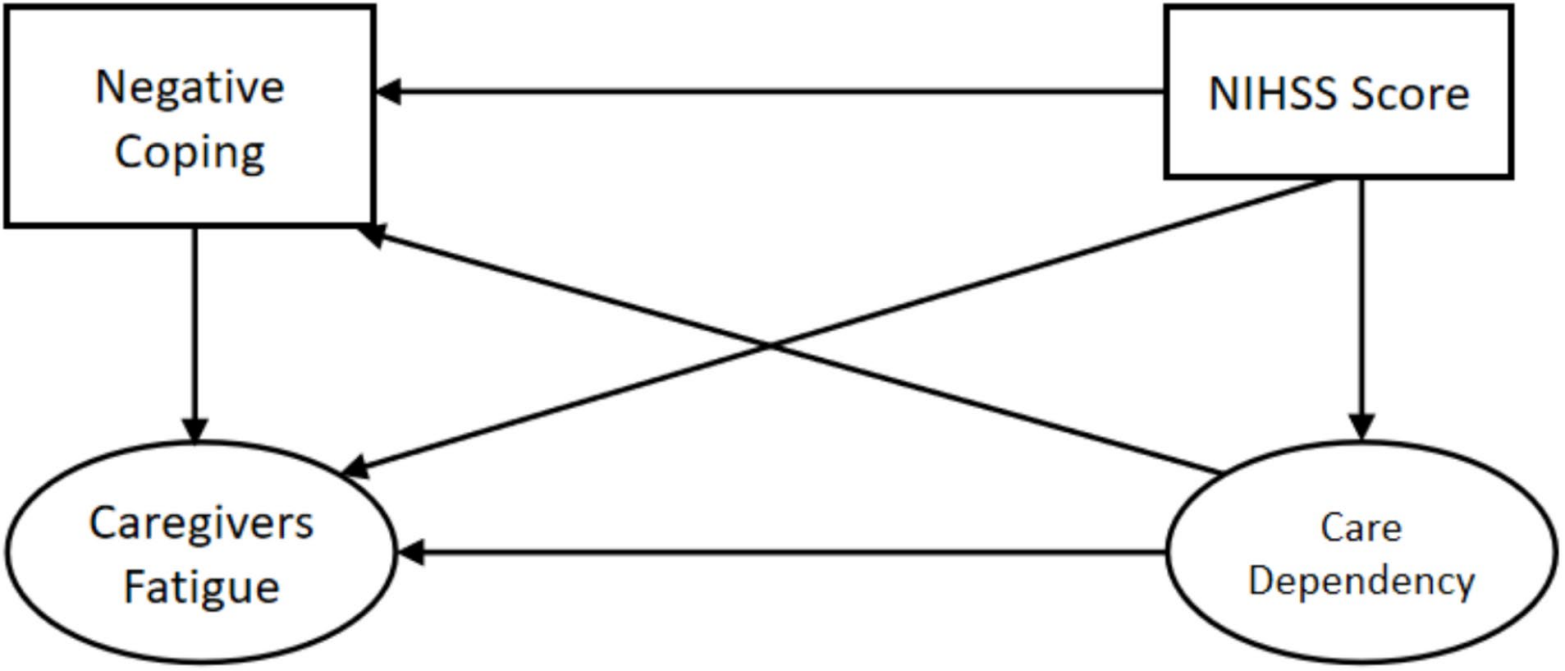
The hypothetical structural equation model of this study.

This study aimed to examine the potential factors related to caring fatigue and their direct and indirect relationship with caregivers of stroke survivors. Based on Lazarus and Folkman’s Transactional Model of Stress and Coping theory, Figure 1 illustrates the hypothetical model that applies to our study. We hypothesized that there would be a positive correlation between the National Institute of Health Stroke Scale (NIHSS) score and caregiver fatigue, as well as between the NIHSS score and care dependency. Additionally, we expected a positive correlation between care dependency and caregiver fatigue. We anticipated that caregivers’ negative coping strategy would impact their level of fatigue. Moreover, we hypothesized that the NIHSS score of patients would indirectly impact caregivers’ fatigue by means of the mediating effects of care dependency and caregivers’ negative coping strategy, and the NIHSS score would also indirectly impact caregivers’ negative coping strategy through the effect of care dependency.

### 3.2 Design, setting, and participants

This was a cross-sectional study enrolling patients in a tertiary general hospital from July 2022 to April 2023 by continuous sampling method. Inclusion criteria: (1) Meeting the clinical diagnostic criteria of acute ischemic stroke and confirmed by brain CT or MRI (Chinese Society of Neurology, 2017); (2) Age ≥18 years; (3) With adequate ability in communication; (4) Informed consent and voluntary participation in this study. Exclusion criteria: (1) Complicated with a malignant tumor and serious disorders of the heart, liver, and kidney; (2) Patients with congenital disabilities or other disabilities. Inclusion criteria for caregivers: (1) Primary caregivers of stroke patients during hospitalization; (2) Age ≥18 years old; (3) With adequate ability in communication; (4) Informed consent and voluntary participation in this study. Exclusion criteria for caregivers: (1) With severe mental disorders, drug or alcohol dependence; (2) Having experienced other major life events in the past 1 month and had obvious psychological stress; (3) Employed caregivers who were paid by patients or their families.

### 3.3 Data collection

Before discharge, trained researchers explained the objectives of this study to potential participants and administered the questionnaire survey through one-to-one interviews after obtaining informed consent forms. This study was approved by the Ethics Committee of XX (Ethics No. 496, 2018). Information was collected for each patient, encompassing demographic data such as age, sex, and Body Mass Index (BMI), as well as stroke-related data, including the NIHSS score for assessing the severity of neurological impairment, the time since stroke onset, comorbidities, and the Care Dependency Scale (CDS) to determine the patients’ level of dependency. A total of 403 dyads of stroke patients and caregivers were eligible and received the questionnaire survey, of whom 380 dyads completed the questionnaire (response rate 94.29%).

### 3.4 Endogenous variables

#### 3.4.1 Care dependency

In this study, we employed the Chinese version of the Care Dependency Scale (CDS) to assess the level of patient needs from caregivers (31). It’s a two-dimensional questionnaire consisting of 14 items, including (1) 8-items assess physiological functions (diet, excretion, body position, daily activity level, dressing and undressing, body temperature, personal hygiene, and avoiding danger); and (2) 6-items measure psychosocial functions (communication skills, networking ability, following morals and values, daily activities, recreational activities, and learning ability). Responses are rated by a 5-point scoring system, ranging from 1 point (complete dependence) to 5 points (complete independence), resulting in a total score range of 14–70. A higher score indicates a lower degree of care dependency. The Cronbach’s α coefficient value of the CDS was 0.976 in this study.

#### 3.4.2 Fatigue of caregivers

We evaluated fatigue using the Fatigue Scale (FS-14) (32), which comprises two dimensions (physical and psychological fatigue) and 14 items, of which items 1–8 and items 9–14 explain physical fatigue and psychological fatigue, respectively. For most items, the answer “Yes” is assigned 0 points, while “No” is 1 point. Items 10, 13, and 14 are scored in reverse. The total FS-14 score ranges from 0 to 14, with a higher score indicating more severe fatigue. In this study, the Cronbach’s α coefficient for FS-14 was 0.86.

#### 3.4.3 Negative coping of the coping strategy

The Simple Coping Style Questionnaire (SCSQ) was adopted to assess the coping strategy of caregivers of stroke survivors. It was designed by Xie (33) in 1998 and contains 20 items, which were divided into two subscales. It measures positive coping style (items 1–12) and negative coping style (items 13–20) by ranking with a 4-point Likert scale; higher scores indicate a higher frequency of adopting the corresponding coping style (34). The SCSQ scale showed good reliability and validity, with the Cronbach’s α coefficient of the positive and negative coping style subscale being 0.9 and 0.85, respectively (33). In this study, the Cronbach’s α coefficient for the positive and negative coping style subscale was 0.92 and 0.77.

### 3.5 Exogenous variables

#### 3.5.1 NIHSS score

The NIHSS scale is a 15-item instrument originally developed in 1989 (35), and it is a widely endorsed instrument for evaluating stroke severity. The total score is calculated by summing the scores from all 15 items, and it ranges from 0 to 42. A higher score reflects a more severe stroke. The NIHSS includes the following nine domains: level of consciousness, eye movements, the integrity of visual fields, facial movements, muscle strength of arms and legs, sensation, coordination, language, speech, and neglect (36).

### 3.6 Statistical analysis

Statistical analysis was conducted using IBM Corp’s SPSS version 22.0 and AMOS version 23.0. The data were described as mean ± standard deviation (SD), and the counting data were interpreted by the frequency, percentage, and composition ratio. The independent *t*-test and one-way analysis of variance (ANOVA) were employed to estimate the differences in fatigue based on the caregivers’ characteristics. The Pearson’s correlation coefficient was utilized to depict the association between variables. A two-tailed *p* < 0.05 was considered statistically significant.

Structural equation modeling (SEM) has proven to be an efficient approach to exploring the inherent relationships among variables. The fit of the model was estimated by the following indices: goodness-of-fit index (GFI), adjusted goodness-of-fit index (AGFI), root mean square error of approximation (RMSEA), nonstandard fit index (NFI), relative fit index (RFI), incremental fit index (IFI), Tucker-Lewis index (TLI), standardized root mean square residual (SRMR), and comparative fit index (CFI). A model with CFI > 0.9, GFI > 0.9, AGFI>0.9, TLI > 0.9, IFI > 0.9, NFI > 0.9, RMSEA<0.08, and SRMR<0.05 was acceptable (37–40). We also performed bootstrapping with the samples of 1,000 to confirm the path coefficients and the significance of direct, indirect, and total effects and reported the 95% bias-corrected interval confidence. The difference is considered statistically significant when *p* values < 0.05 (41).

## 4 Results

### 4.1 Sample characteristics

As shown in Table 1, the sample comprised 380 dyads. The mean age of stroke survivors was 64.07 ± 12.62 years, and their caregivers had a mean age of 63.41 ± 12.87 years. More than half of the stroke survivors (51.3%) and caregivers (53.4%) were aged 66 or older. Among the participants, there were 257 male patients (67.6%) and 256 female caregivers (67.4%). The majority of both patients and caregivers were unemployed with primary or secondary educational levels. Table 1 also depicts variations in caregivers’ fatigue scores across different demographic characteristics. Among the 380 stroke caregivers, those who were married (97.4%) and devoted more than 12 h a day to caregiving tasks (17.9%) reported higher fatigue scores. Caregivers who perceived their health status and sleep status as poor exhibited more severe fatigue. Additionally, 56 caregivers (14.8%) with more than two co-caregivers experienced higher fatigue scores, indicating severe fatigue levels.

**Table 1:** Baseline characteristics of stroke patients and their family caregivers and differences in caregivers’ fatigue (*N* = 380 dyads).

Characteristics	Patients		Caregivers			
	*n*	%	*n*	%	Fatigue Mean (SD)	*t*/*F* (*p*)
Sex						
Male	257	67.6	124	32.6	8.19 ± 3.82	−0.56 (0.574)
Female	123	32.4	256	67.4	8.41 ± 3.47	
Age						
≤44	21	5.5	25	6.6	8.28 ± 2.73	2.76 (0.065)
45 ~ 65	156	41.1	160	42.1	7.86 ± 3.48	
≥66	203	53.4	195	51.3	8.75 ± 3.72	
Marital status						
Married	333	87.6	370	97.4	8.42 ± 3.56	2.56 (0.011)
Unmarried	47	12.4	10	2.6	5.5 ± 3.57	
Education						
Primary education	153	40.3	130	34.3	8.56 ± 3.41	0.6 (0.55)
Secondary education	179	47.1	205	53.9	8.16 ± 3.52	
Higher education	48	12.6	45	11.8	8.56 ± 4.34	
Monthly household income (¥)						
≤3,000	104	27.4	189	49.7	8.54 ± 3.69	0.68 (0.506)
3,001 ~ 6,000	167	43.9	161	42.4	8.19 ± 3.42	
≥6,001	109	28.7	30	7.9	7.9 ± 3.75	
Working situation						
Workless	273	71.8	314	82.6	8.51 ± 3.67	2 (0.053)
Employed	107	28.2	66	17.4	7.55 ± 3.05	
Perceived health status						
Good	NA	NA	334	87.9	7.95 ± 3.54	−8.14 (<0.01)
Poor	NA	NA	46	12.1	11.2 ± 2.36	
Perceived sleep status						
Good	NA	NA	286	75.3	7.54 ± 3.58	−10.42 (<0.01)
Poor	NA	NA	94	24.8	10.79 ± 2.22	
Caregivers’ role						
Spouse	NA	NA	213	56.1	8.36 ± 3.41	0.68 (0.509)
Children	NA	NA	130	34.2	8.15 ± 3.87	
Others*	NA	NA	37	9.7	8.92 ± 3.53	
Number of co-caregivers						
0	NA	NA	246	64.7	7.7 ± 3.42	13.72 (<0.01)
1	NA	NA	78	20.5	9.05 ± 3.4	
≥2	NA	NA	56	14.8	10.18 ± 3.75	
Daily hours for caring						
≤12 h/day	NA	NA	312	82.1	8.15 ± 3.68	−2.58 (0.011)
>12 h/day	NA	NA	68	17.9	9.22 ± 2.96	

*Others refer to patients’ grandchildren, brothers, sisters, nieces, nephews, etc.; NA, not available.

### 4.2 Mean scores and correlations of the study variables

In this study, the mean score for the NIHSS score, care dependency, fatigue scale, positive coping, and negative coping were 6.79 ± 5.09, 53.91 ± 15.95, 8.34 ± 3.58, 19.39 ± 7.05, and 8.06 ± 4.23, respectively. The care dependency score, CD-physiological functions score, and CD-psychosocial functions score were all negatively correlated with the FS score, FS-physical fatigue, FS-psychological fatigue, and negative coping score. However, no significant correlation was found between the CD-psychosocial functions score and FS-psychological fatigue score (*r* = −0.088, *p >* 0.05). The NIHSS score was negatively associated with the CD score, CD-physiological functions score, and CD-psychosocial functions score while demonstrating a positive correlation with the FS score, FS-physical fatigue score, and negative coping score. Furthermore, we observed positive associations between the FS-physical fatigue score and negative coping score, as well as between the FS score and positive coping score. For further details on mean scores, standard deviation across different variables, and their respective Pearson’s coefficients, refer to Table 2.

**Table 2:** Mean scores and correlations for NIHSS score, care dependency, fatigue, and coping strategy between stroke caregivers.

Variables	Means (SD)	*r* value							
		1	2	3	4	5	6	7	8
1. NIHSS score	6.79 (5.09)	1							
2. CD-physiological functions	30.45 (9.47)	−0.637**	1						
3. CD-psychosocial functions	23.46 (6.91)	−0.634**	0.892**	1					
4. CD score	53.91 (15.95)	−0.635**	0.981**	0.963**	1				
5. FS-physical fatigue	5.47 (2.20)	0.238**	−0.34**	−0.277**	−0.322**	1			
6. FS-psychological fatigue	2.87 (1.79)	0.094	−0.132**	−0.088	−0.117*	0.605**	1		
7. FS score	8.34 (3.58)	0.193**	−0.275**	−0.214**	−0.256**	0.917**	0.872**	1	
8. Positive coping	19.39 (7.05)	0.017	−0.08	−0.063	−0.075	0.084	0.099	0.102*	1
9. Negative coping	8.06 (4.23)	0.224**	−0.319**	−0.27**	−0.307**	0.139**	−0.019	0.076	0.186**

CD, care dependency; FS, fatigue scale; SD, standard deviation; NIHSS, National Institute of Health stroke scale; **p < 0.01; *p < 0.05.

### 4.3 Testing the research hypothesis and analyzing the parameter estimates

**Figure 2:**
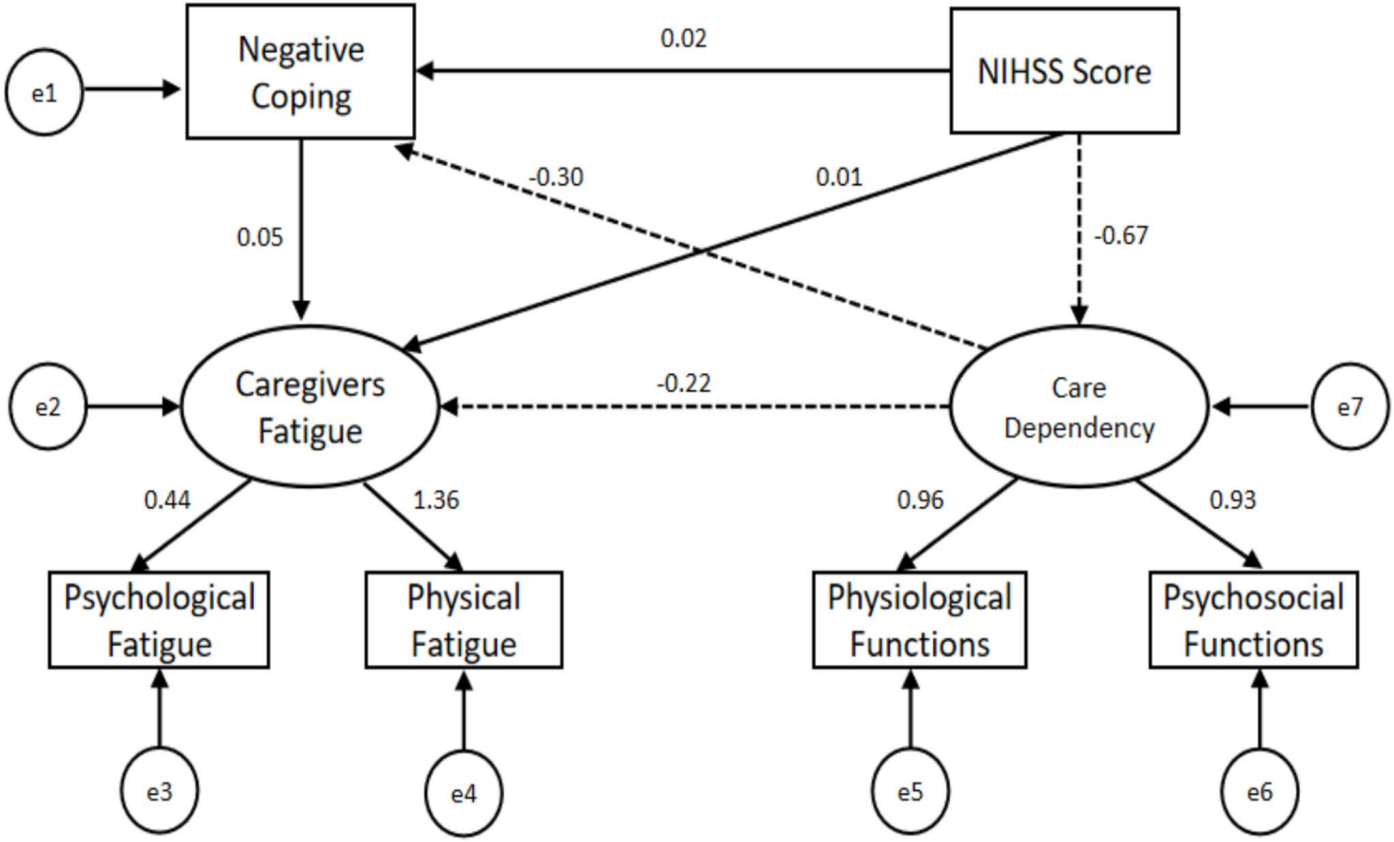
The details of the standardized coefficients.

The results of the hypothetical model exhibited a good fit to the data with χ^2^ = 12.595 (*df* 5), IFI = 0.993, GFI = 0.990, AGFI = 0.956, NFI = 0.988, TLI = 0.979, CFI = 0.993, RMSEA = 0.063, SRMR = 0.0213. Among the 11 pathways within this hypothetical model, we confirmed that seven of them revealed statistically significant direct, indirect, or total effects. Specifically, the direct effect path coefficient of the NIHSS score on patients’ care dependency was −0.67 (*p* = 0.002), while registering 0.01 (*p* = 0.832) on caregivers’ fatigue and 0.02 (*p* = 0.693) on negative coping. The direct effect path coefficient of patients’ care dependency on caregivers’ fatigue (β = −0.22, 95% CI: −0.367, −0.097, *p* = 0.002) and negative coping (β = −0.31, 95% CI: −0.438, −0.155, *p* = 0.003) was statistically significant. Notably, caregivers’ negative coping strategy did not exhibit a significant direct effect on their fatigue. Importantly, the NIHSS score indirectly influenced caregivers’ fatigue (β = 0.023, 95% CI: 0.006, 0.051, *p* = 0.002) and negative coping (β = 0.204, 95% CI: 0.103, 0.303, *p* = 0.003) through the effect of care dependency. However, caregivers’ negative coping strategy did not serve as the mediator between patients’ care dependency and caregivers’ fatigue (β = −0.015, 95% CI: −0.043, 0.006, *p* = 0.136). The direct, indirect, and total effects are presented in Table 3, while Figure 2 illustrates the details of the standardized coefficients.

**Table 3:** The direct, indirect, and total effect for NIHSS score, care dependency, fatigue, and coping strategy between stroke caregivers.

Endogenous variables	Exogenous variables	Pathway	SE	CR	*p*	Direct effect		Indirect effect		Total effect	
						β (*p*)	95% CI	β (*p*)	95% CI	β (*p*)	95% CI
1. Caregivers fatigue	2. NIHSS score	2 → 1	0.007	0.277	0.781	0.013 (0.832)	(−0.098, 0.098)			0.170 (0.003)	(0.082, 0.270)
		2 → 4 → 1						0.001 (0.438)	(−0.001, 0.003)		
		2 → 3 → 4 → 1						0.002 (0.119)	(−0.001, 0.005)		
		2 → 3 → 1						0.023 (0.002)	(0.006, 0.051)		
1. Caregivers fatigue	3. Care dependency	3 → 1	0.01	−1.974	0.048	−0.22 (0.002)	(−0.367, −0.097)			−0.233 (0.002)	(−0.377, −0.111)
		3 → 4 → 1						−0.015 (0.136)	(−0.043, 0.006)		
1. Caregivers fatigue	4. Negative coping	4 → 1	0.008	1.154	0.249	0.048 (0.163)	(−0.022, 0.123)			0.048 (0.163)	(−0.022 0.123)
3. Care dependency	2. NIHSS score	2 → 3	0.073	−16.43	***	−0.669 (0.002)	(−0.747, −0.584)			−0.669 (0.002)	(−0.747, −0.584)
4. Negative coping	2. NIHSS score	2 → 4	0.056	0.307	0.759	0.021 (0.693)	(−0.105, 0.148)			0.224 (0.002)	(0.132, 0.311)
		2 → 3 → 4						0.204 (0.003)	(0.103, 0.303)		
4. Negative coping	3. Care dependency	3 → 4	0.032	−4.405	***	−0.31 (0.003)	(−0.438, −0.155)			−0.305 (0.003)	(−0.438, −0.155)

## 5 Discussion

Our study aimed to develop and validate a structural equation model, assessing the connections among patients’ care dependency, NIHSS score, caregivers’ negative coping strategy, and caregivers’ fatigue and thoroughly exploring potential mediating effects among these variables. Although the structural equation model had a good fit index with χ^2^ = 12.595 (*df* 5), IFI = 0.993, GFI = 0.990, AGFI = 0.956, NFI = 0.988, TLI = 0.979, CFI = 0.993, RMSEA = 0.063, SRMR = 0.0213. Our model is nearly saturated with only 5 degrees of freedom, limiting its capacity to reflect true model fit. However, adopting SEM is still valuable when modeling latent variables or complex multivariate relationships beyond the scope of simpler regression or mediation analyses.

In this study, the mean age of the stroke survivors was older than that of participants in a Chinese national cohort-based study conducted in 2013, while their educational level was consistent with that of the aforementioned participants (42). It may be a result of population aging over time. As life expectancy extends and the population ages, it is anticipated that the mean age of individuals affected by stroke will notably rise, paralleling the age range of their caregivers (11). It is well-known that stroke caregivers dedicate a substantial amount of time to their role (43, 44), and the more caring tasks associated with caregiver burden and fatigue, the more time caregivers spend on the caregiving tasks, the less time was left for caregivers to rest subsequently. Menon et al. (45) found that a good portion of caregivers suffered from sleep disturbance, even insomnia, due to the multiple responsibilities and incontinence in patients with nocturnal awakenings. The consequence of sleep disturbance, in turn, affects caregivers’ health, which would trigger a cycle of fatigue and exhaustion (46, 47). Interestingly, we found that caregivers with more than two co-caregivers reported higher fatigue or burden, contrary to those caregivers who co-cared with foreign domestic workers in a study conducted in Singapore (48). From the norms of Confucian ideology (e.g., filial piety) and familism of traditional culture, the Chinese caregiver role may be usually played by family members, close relatives, or friends. Deeply rooted Chinese cultural traditions often compel family caregivers to undertake undesirable caregiving tasks. Among co-caregivers, it is common for some to pay attention to the efforts and contributions made by other family caregivers, especially close relatives (49, 50). The inherent tension and conflicts (including financial responsibilities, distribution of caregiving hours, and social isolation) can breed dissatisfaction and mood swings throughout the caregiving trajectory, which may lead to not only physical fatigue but also psychological fatigue for caregivers.

The mean score for the care dependency, fatigue scale, positive coping, and negative coping of our study were consistent with other findings of the Chinese population (51). The NIHSS score is a widely used instrument to assess stroke-related neurological deficits and outcome severity (52, 53). It was recommended as a standard in nursing care to monitor the severity of neurological functions, and it was accurate in predicting the patients’ ADL dependency (54). Stroke patients with a higher NIHSS score (indicating a worse neurological status) tend to be more dependent. Our results showed that the NIHSS mean score for the stroke survivors is 6.75, indicating that they were in moderate stroke severity with partial activities of daily living (ADL) (55). As compared to the general population, stroke patients with impaired physical function and a worse NIHSS score affect multiple facets of their lives. Dewilde et al. (30) indicated that the stroke survivors’ care dependency was closely linked to survivors’ mobility (unable to walk without assistance, being in a wheelchair, being bedridden) and the resulting need for both physical and psychological assistance from others.

Stroke caregivers, often unprepared for their caregiving responsibilities, are immediately entrusted with managing patients’ hygiene care, monitoring health and illness, administering medications, planning and coordinating social activities, and managing finances (56). They are exposed to various care dependency and caregiving stressors, encompassing physical fatigue, financial strain, social isolation, and a myriad of mental and emotional stress, which imposes a significant burden. They have fallen victim to care burden and fatigue originating from the prolonged and intense caregiving experience (57). Caregivers’ fatigue is not only caused by excessive physical activities but also by overwhelming mental burdens, which leads to inimitable discomfort and a condition of decreased physical activity with a desire for rest (58).

Over time, a sense of fatigue may become increasingly apparent if effective coping mechanisms are not employed. It is reported that as many as 75% of stroke caregivers report unmet needs and concerns during the first few months at home because of a lack of training and ability to provide care, especially when caregivers have insufficient physical and psychological health to cope with those unmet needs (59). They tend to adopt negative coping strategies when they perceive that the patient’s care needs exceed their caregiving capabilities and they are unwilling or unable to seek support for their own needs (10, 60), which would result in physical and psychological exhaustion, and may even contribute to burnout. Getting used to changing lives and trying to manage fatigue for stroke caregivers is of great importance. Some caregivers suggested accepting the impact of fatigue on their lives and the required ensuing changes instead of fighting for it or coping negatively (23). Caregivers often want to participate in the recovery process, providing tangible assistance and support to stroke survivors (61). The bond of affection and aspirations for the recovery of loved ones is the inner motivation for caregivers to employ positive coping strategies. Mou et al. (62) found that dyadic interventions, which focus on establishing shared illness appraisals and collaborative illness management from the perspective of a mutually supportive relationship between stroke survivors and their caregivers, would have satisfactory outcomes. A systematic review also recommended combining skill-building and psychoeducational strategies to improve the well-being of stroke caregivers (63). Additionally, some interventions underpinned by electronic assistive technologies, such as health watches, interactive artificial intelligence systems, and telehealth systems, hold great promise to support stroke caregivers (64). These positive coping strategies can assist caregivers in alleviating caregiving distress, reducing the post-stroke fatigue, and cutting healthcare costs.

## 6 Conclusion

Post-stroke fatigue is prevalent among caregivers of stroke survivors and remains inadequately investigated, making it difficult to formulate definitive conclusions on its management. The primary approach explored in this study to cope with stroke caregivers’ fatigue highlights the importance of optimizing the recovery process to alleviate patients’ care dependency. Supports from a dyadic perspective are expected to benefit both counterparts of the dyad by treating both as active participants while considering mutualistic relationships and interactions between caregivers and stroke survivors. Moreover, interventions concentrate on enabling caregivers to adopt more positive coping strategies, such as confronting or problem-focused coping, which may be an effective means to encourage them to sustain their caregiving role as long as possible without compromising their well-being.

## 7 Limitation

This study has several limitations. First, although this study was the largest study in China to investigate the fatigue status of stroke caregivers, it was conducted in a single hospital, which needs to be validated in multicenter settings. Secondly, the cross-sectional nature of this study could not trace the dynamic impact of caregivers’ fatigue on the rehabilitation period of stroke survivors. A trajectory needs to be investigated further in future longitudinal studies. Thirdly, only negative coping pathways were included in the final SEM analysis. This might not be sufficient to comprehensively explain the interrelationships among the stroke survivors’ care dependency, fatigue, and coping strategies in their caregivers. Expanding the sample size and increasing its diversity in subsequent investigations could potentially solve this problem. Lastly, examining fatigue and its determinants during hospitalization was restricted. There is an urgent need to extend relevant studies involving a broader range of influencing variables to the post-discharge period. Understanding the stroke caregivers’ fatigue change, which integrates the hospitalization and post-discharge phases throughout the comprehensive rehabilitation process, is crucial for the development of targeted interventions to cope with the care burden.

## Data Availability

The raw data supporting the conclusions of this article will be made available by the authors, without undue reservation.
